# Systematic review and assessment of systematic reviews examining the effect of periodontal treatment on glycemic control in patients with diabetes

**DOI:** 10.4317/medoral.21555

**Published:** 2017-02-04

**Authors:** Akira Hasuike, Shinya Iguchi, Daigo Suzuki, Eisuke Kawano, Shuichi Sato

**Affiliations:** 1 DDS, PhD. Assistant Professor. Department of Periodontology, Dental Research Center, Nihon University School of Dentistry; 2 DDS. Graduate student. Division of Applied Oral Sciences, Nihon University Graduate School of Dentistry; 3 DDS, PhD. Professor. Department of Periodontology, Dental Research Center, Nihon University School of Dentistry

## Abstract

**Objetives:**

There have been several systematic reviews(SRs) on whether periodontal treatment for an individual with both periodontal disease and diabetes can improve diabetes outcomes. The purpose of this investigation was to conduct a systematic review (SR) of previous meta-analyses, and to assess the methodological quality of the SRs examining the effects of periodontal treatment and diabetes. (PROSPERO Registration # CRD 42015023470).

**Study Design:**

We searched five electronic databases and identified previous meta-analyses of randomized controlled trials published through July 2015. In cases where the meta-analysis did not meet our criteria, the meta-analyses were recalculated. General characteristics of each included trial were abstracted, analyzed, and compared. The mean difference, 95% confidence intervals (CIs) and the I2 statistic were abstracted or recalculated. The Assessment of Multiple Systematic Reviews Instrument (AMSTAR) was used to assess methodological quality.

**Results:**

Of the 475 citations screened, nine systematic reviews were included. In total, 13 meta-analyses included in nine SRs were examined. In comparability analyses, meta-analyses in four SRs did not meet our criteria, and were recalcuated. Of these 13 meta-analyses, 10 suggested significant effects of periodontal treatment on HbA1c improvement. Mean differences found in the 13 meta-analyses ranged from -0.93 to 0.13. AMSTAR assessment revealed six SRs with moderate and three with high overall quality.

**Conclusions:**

We can conclude that there is a significant effect of periodontal treatment on improvement of HbA1c in diabetes patients, although the effect size is extremely small. In addition to the small effect size, not all SRs could be considered of high quality.

**Key words:**Periodontal treatment, diabetes, HbA1c, systematic review, systematic review of systematic reviews, evidence-based medicine, AMSTAR.

## Introduction

Periodontal disease is one of the most prevalent chronic infections in adults worldwide. Many researchers have explored the association between periodontal diseases and diabetes over the years. Studies have shown that diabetic patients have a 2-3-fold higher risk of developing severe periodontitis and progressive periodontal disease (1). There has been much emphasis on the two-way relationship between periodontal disease and diabetes. Diabetes has many adverse effects on periodontal tissue, and conversely, periodontitis may further aggravate the diabetic condition. One of major questions in this two-way relationship is whether periodontal treatment in an individual with both periodontal disease and diabetes can improve diabetes outcomes.

Although clinical trials are a basis for good evidence, there is not always time to search for, read, and evaluate many primary studies. It is much easier to find and read a summary or review of the evidence. Systematic reviews (SRs) aim to assimilate high-quality evidence in an area of interest in a systematic, transparent, and unbiased manner, leading to a qualitative or quantitative synthesis. Multiple SRs may have been conducted on clinical questions that interest many clinicians. As the number of SRs begins to grow, one is likely to find different SRs on the same topic, conducted with different aims and methodologies, and sometimes leading to conflicting results. Thus, critical reading and evaluation are necessary not only in assessing clinical trials but also in assessing SRs. Consequently, there is a need for efforts to provide an overview and comparison of existing SRs in a single paper.

 We aimed to systematically review existing SRs and to summarize the evidence relating to the effects of periodontal treatment on diabetes outcomes. Thus, the aims of the present study were

• To provide an overview of the reported effects of periodontal treatment on diabetes outcomes and to rate the evidence on which these effects are based; and

• To assess the methodological quality of the SRs examining the effects of periodontal treatment and diabetes.

## Material and Methods

- Study Eligibility

This systematic review of previous systematic reviews of meta-analyses is registered in the PROSPERO trial registry (CRD 42015023470). Given that no guidelines currently exist for conducting SRs of previous SRs, the general guidelines of the Preferred Reporting Items for Systematic Reviews and Meta-Analysis (PRISMA) Statement, where applicable, were followed (2). Following Smith *et al.’s* approach for conducting a SR of SRs in healthcare interventions, the participants, interventions, comparisons, outcomes, and study design (PICOS) structure is recommended (3). Thus, the scope of our topic can be rewritten according to the PICO structure as follows:

P: Patients with type 1 or type 2 diabetes diagnosed with periodontitis, regardless of the classification.

I: Periodontal treatment with or without adjunctive use of local drug delivery and systemic antibiotics.

C: Control group with no periodontal treatment or delayed treatment.

O: Changes in glycated hemoglobin (HbA1c).

 Inclusion criteria for this study were: previous systematic reviews of meta-analyses of clinical trials; participants 16 years of age and older with type 1 or type 2 diabetes and periodontitis; interventions consisting of periodontal treatment with or without adjunctive use of local drug delivery and systemic antibiotics; control group with no periodontal treatment or delayed treatment; study duration more than 3 months; and reporting data about glycated hemoglobin (HbA1c).

Studies that did not meet all of the criteria were excluded. Ineligible studies were excluded based on one or more of the following: inappropriate populations (for example, children); inappropriate interventions (for example, periodontal treatment less than 3 months); inappropriate comparisons (for example, comparison among different kind of periodontal treatment); inappropriate outcomes (for example, serum levels of interleukin or CRP); and inappropriate study types (for example, systematic review without meta-analysis).

- Search Strategy

Five databases were searched: MEDLINE (PubMed) (1966 to July 25, 2015); Web of Science (1955 to July 25, 2014); Cochrane Database of Systematic Reviews (1996 to July 25, 2015); Trip Database (to July 25, 2015); and Centre for Reviews and Dissemination Database (1960 to July 25, 2014). We combined search terms and limited the search to humans and the English language. While the specific search strategies varied depending on the database searched, key terms or forms of key terms included diabetes, periodontal, systematic review and meta-analysis, using identical search criteria and terms: ((periodontal disease) OR (periodont*[Text Word]) OR (periodontitis) AND (diabetes[Text Word]) OR (diabet*[Text Word]) OR (diabetic*[Title]) OR (diabetic patient*[Text Word]) OR (diabetes patient[Text Word]) OR (non-insulin-dependent diabetes) OR (niddm[Text Word]) OR (insulin dependent diabetes[Text Word]) OR (iddm[Text Word]) OR (type 1 diabetes) OR (t1 dm) OR (type 2 diabetes) OR (t2 dm) AND (therapy) OR (treatment) OR (intervention)) AND systematic[sb] AND (english[Language]). In addition, manual searches of the references from selected original research and review articles were also conducted.

- Comparability Analysis of Meta-analyses

There were various forms and patterns of meta-analyses in the included SRs. Because it is difficult to compare results of meta-analyses described in various formats, it was necessary to unify the forms of the meta-analyses. Consequently, prior to data synthesis, the following four criteria were verified. In cases where the meta-analysis did not meet any of these four criteria, the meta-analyses were recalculated.

• Are outcome indices presented as comparisons of %A1C improvement between the intervention and the control groups?

• Are there any obvious transcription errors between indices presented in the meta-analysis and original trials?

• Is a random effects model used for combining data in the meta-analysis?

• Are results of a heterogeneity analysis presented?

Meta-analyses that failed any of these items needed to be recalculated. In these cases, the Revman5.3 software was used to generate meta-analyses from indices presented in reports of the original trials. Data from the regenerated meta-analyses were then used in our analysis.

- Data Analysis

 General characteristics of each included trial were abstracted, analyzed, and compared. The mean difference and 95% confidence intervals (CIs) were abstracted or recalculated. The I2 statistic, a measure of heterogeneity, was also abstracted if it was provided in the meta-analysis. If I2 was not reported, it was calculated, if sufficient data were available.

Methodological Quality assessment: AMSTAR checklist

The proliferation of systematic reviews in the clinical field renders it challenging for clinicians to use reviews in making clinical decisions because it is difficult to distinguish good from poor-quality reviews; the AMSTAR checklist is an easy-to-use tool purposely developed to address this need (4). Two reviewers (SI, EK) independently rated study quality using the 11-item AMSTAR checklist; where differences were noted, these were resolved by discussion between the two reviewers, and where agreement could not be reached, a third reviewer (DS) resolved the issue. Finally, the score for each item on the checklist was analyzed individually (AMSTAR matrix analyzed by columns) to identify which items future research should focus on to improve the quality of reviews.

## Results

- Study Selection

**Figure 1:**
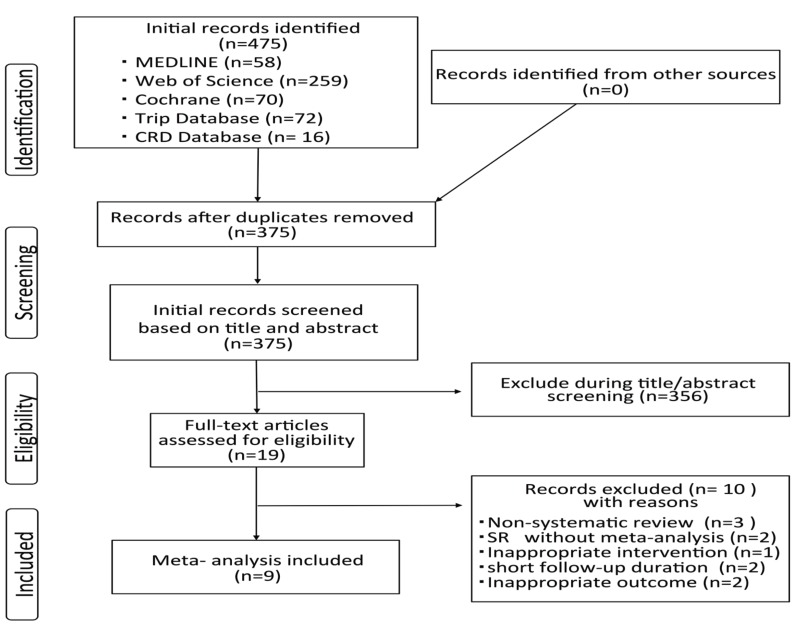
Flow diagram for the selection of SRs.

In total, 475 references were initially identified. After duplicate were removed, 375 articles remained. Of the 375 screened, 19 articles were selected for final eligibility assessment. Of the 19 articles, nine met all study eligibility criteria (5-13). Figure 1 illustrates the search process.

Characteristics of Included SRs

First, comparability analyses of the meta-analyses were conducted. The meta-analyses in four SRs did not meet our criteria ( Table 1). Obvious transcription errors between indices presented in the meta-analysis or original trials were confirmed in three of four SRs. In Simpson’s Cochrane review, the index was presented as a comparison of HbA1c measured after following up between test and control (12), although in others, it was presented as comparison of the two groups in in HbA1c improvement. We recognized the need to include these four meta-analyses, and we present our newly calculated meta-analyses. In the meta-analysis of Simpson’s Cochrane review article, recalculation was done the improvement in HbA1c in both groups and a random effects model. The reanalyzed meta-analysis revealed that the newly calculated 95% CI range was wider than that reported in the original SR and that there was no statistically significant difference between the groups. Thus, this result was inconsistent with that reported in the original meta-analysis.

**Table 1:**
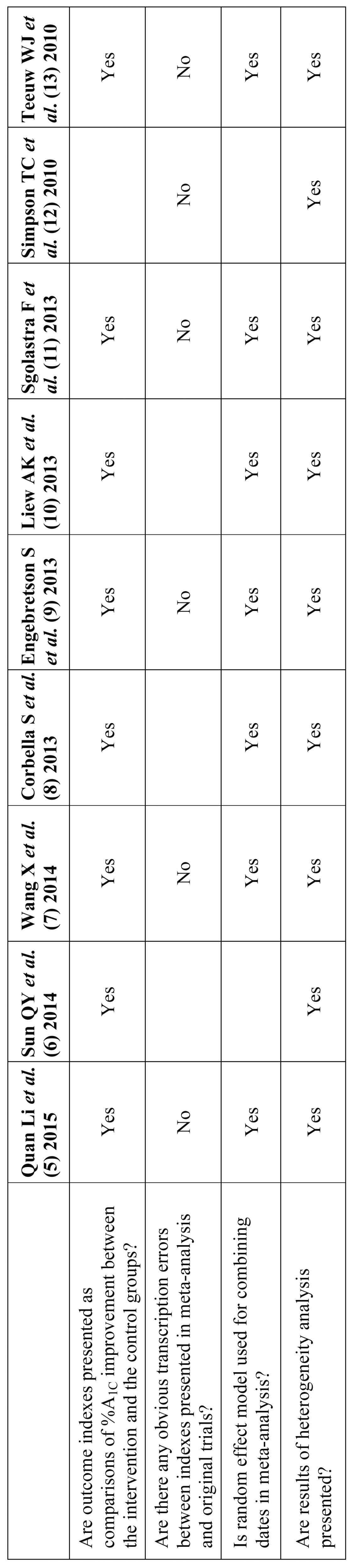
Results of comparability assessment of included SRs.

General characteristics of the nine SRs included are described in  Table 2 and 2 continue. Although minor differences existed in the details of the periodontal treatment, no fatal inconsistency relative to the PICO framework was observed in the nine SRs.

**Table 2:**
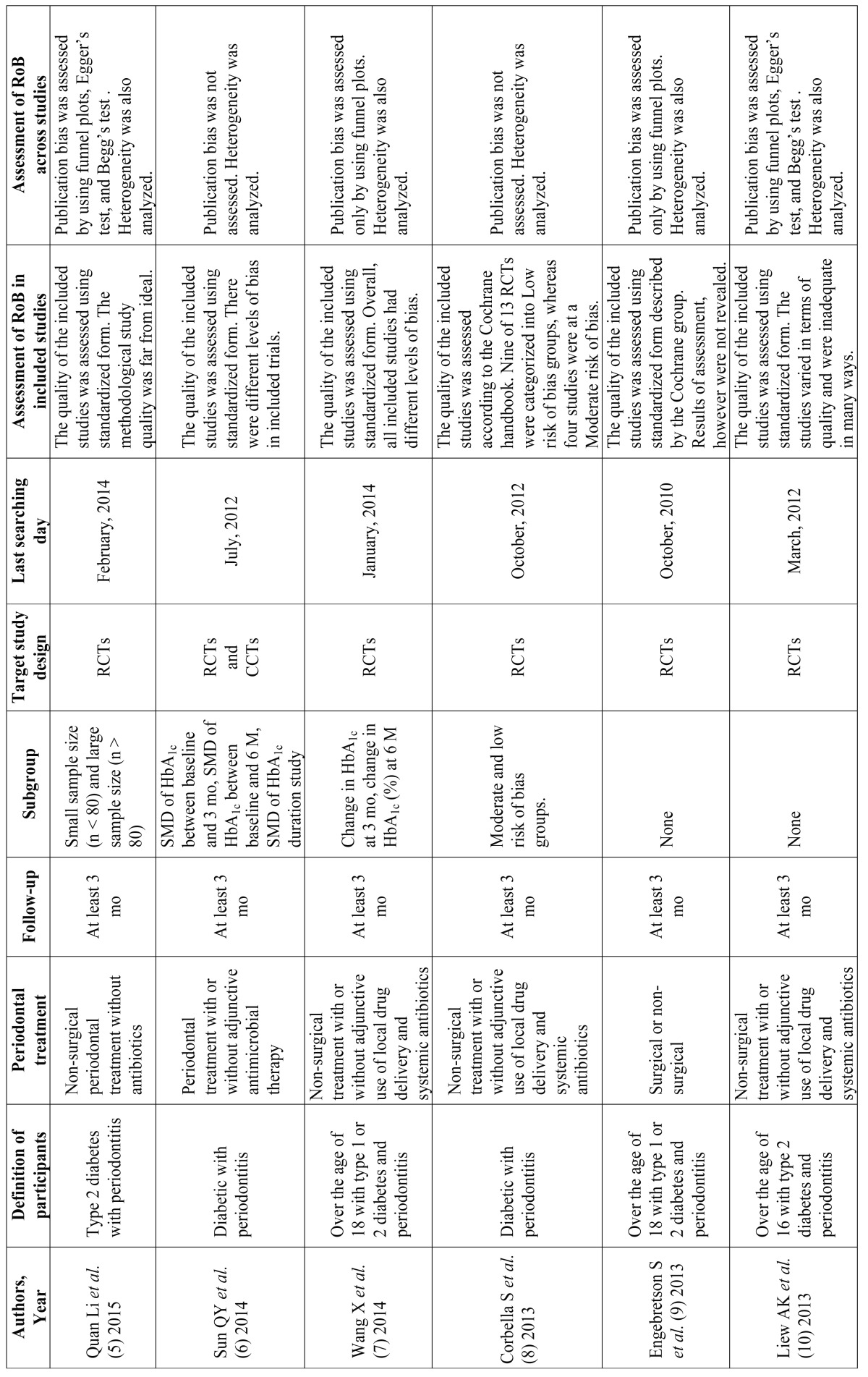
General characteristics of included SRs.

**Table 2 continue:**
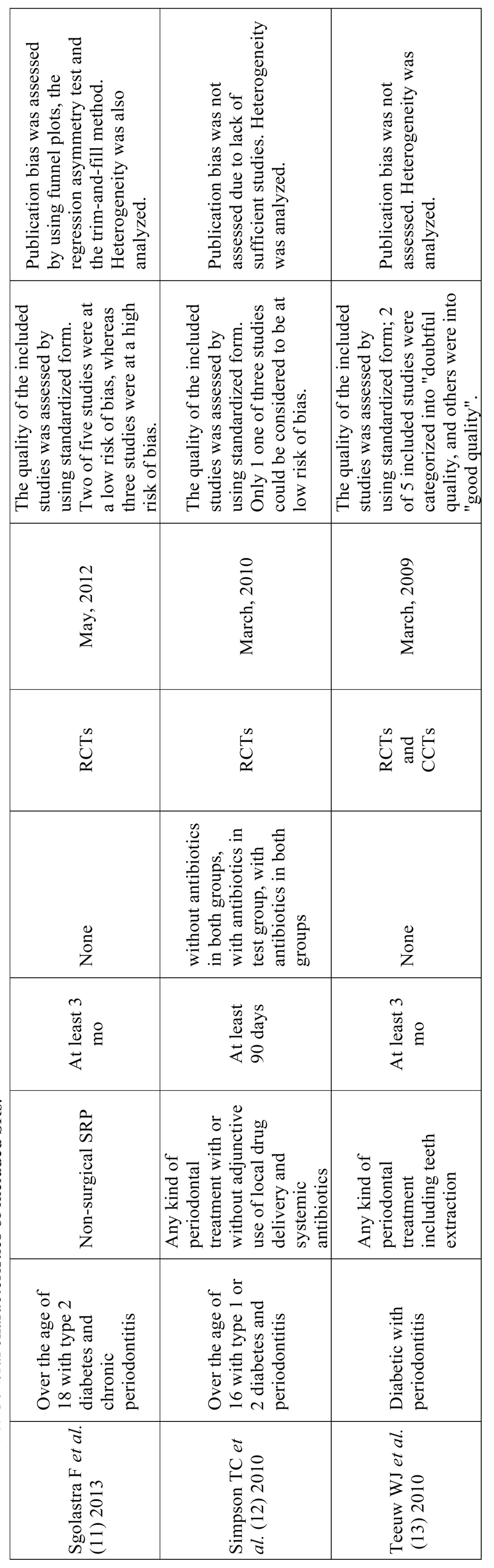
General characteristics of included SRs.

Two of the nine SRs included not only randomized controlled trials but also clinical controlled trials (6,13). The last search day was in 2012 in four of the nine SRs (6,8,10,11). There were several SRs that conducted sub-group analyses focused on sample size (5), follow-up period (6,7), risk of bias (RoB) (8), and use of antibiotics (12).

A quality assessment of each included clinical trial was made for all nine SRs using some type of risk-of-bias tool. For example, sequence generation, allocation concealment, blinding, incomplete outcome data, and selective outcome reporting were assessed. An analysis of publication bias was conducted in five of nine SRs (5,7,9-11), and an analysis of heterogeneity was conducted in all nine SRs (Table 2 and 2 continue).

In total 25, clinical trials were included in the nine SRs. Of the 25 trials, 12 were included in more than two SRs (14-25). Only one trial was included in all nine SRs (14).

Efficacy of periodontal treatment

There were three categories of follow-up time among the included meta-analyses: 3-month duration, 6-month duration, and all durations combined. In total, 13 meta-analyses were reported in the nine SRs. Of the 13 meta-analyses, 10 suggested significant improvement in HbA1c by periodontal treatment. The values of mean differences suggested in the 13 meta-analyses ranged from -0.93 to 0.13 ( Table 3). Results of the comparison of the 13 meta-analyses indicated a significant effect of periodontal treatment on improved HbA1c in diabetes patients, although the effect size was extremely small. Nevertheless, even this small improvement in HbA1c must be interpreted with care due to high heterogeneity, as evidenced by I2 values over 40% confirmed in nine of the 13 meta-analyses.

**Table 3:**
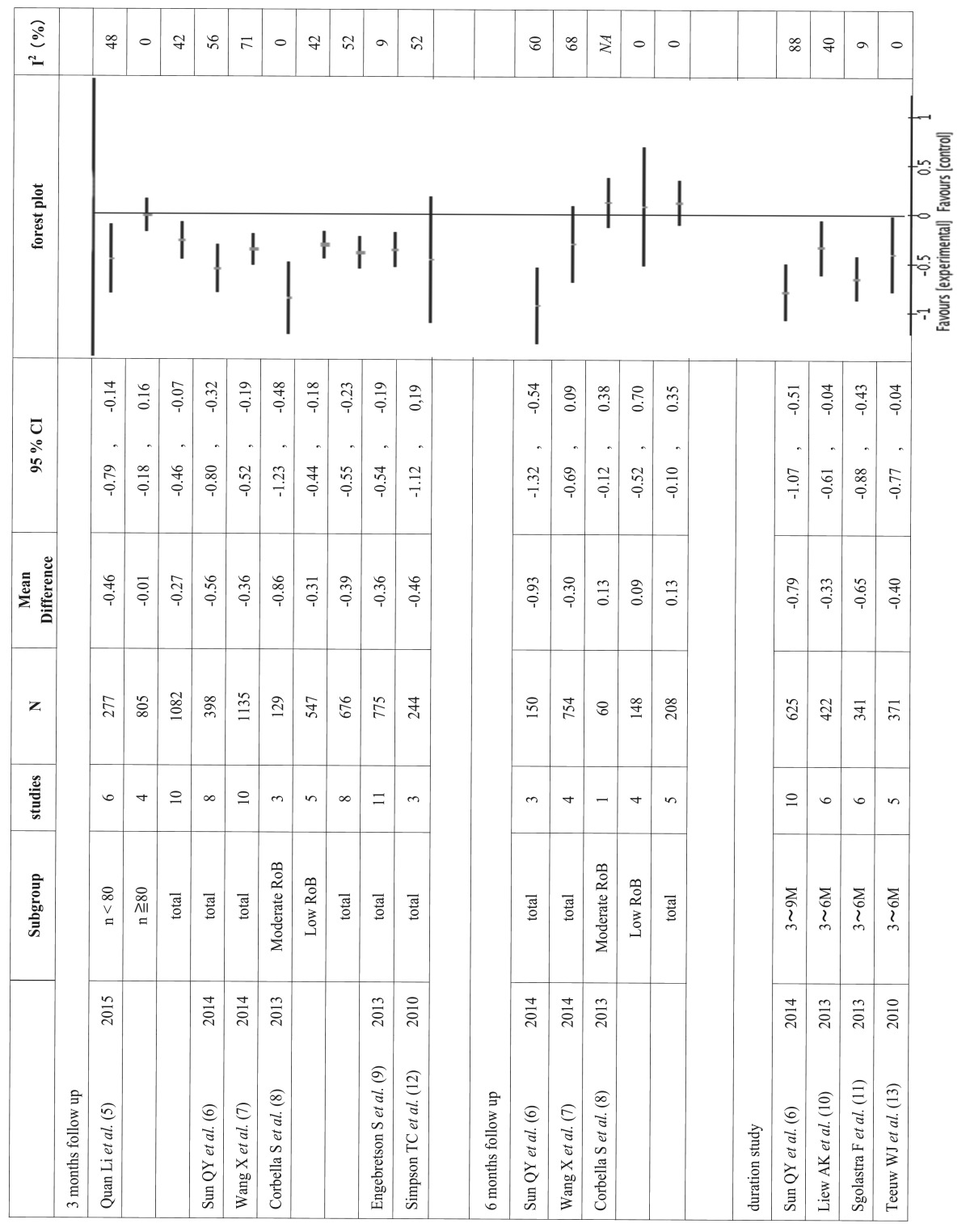
Overall mean differences and forest plots of included meta-analyses.

In a comparison between meta-analyses with a 3-month and those with a 6-month follow-up, no correlation between observation period and effect size was seen. In a sub-group analysis, Quan Li *et al.* stratified the analysis by sample size (5). Their analysis suggested that subgroup analysis of small RCTs (n <80) showed a greater effect size and smaller heterogeneity than did large sample size trials.

- AMSTAR Assessment

Assessment of the methodological quality of the selected reviews showed that no review answered all 11 questions of the AMSTAR tool. Overall, the AMSTAR tool revealed six studies with moderate (four to seven) and three with high (eight to eleven) methodological quality ( Table 4). Reviews that stood out from the rest were the SRs published by Sgolastra *et al.*(11) and by Simpson *et al.* (12) These SRs reported both the included and the excluded studies in the publication (item 5). All of the included SRs had a focused question (item 1) and provided characteristics of the included studies in the form of tables (item 6). None of the nine SRs reported conflicts of interest for not only the SRs themselves but also each trial included in the SRs.

**Table 4:**
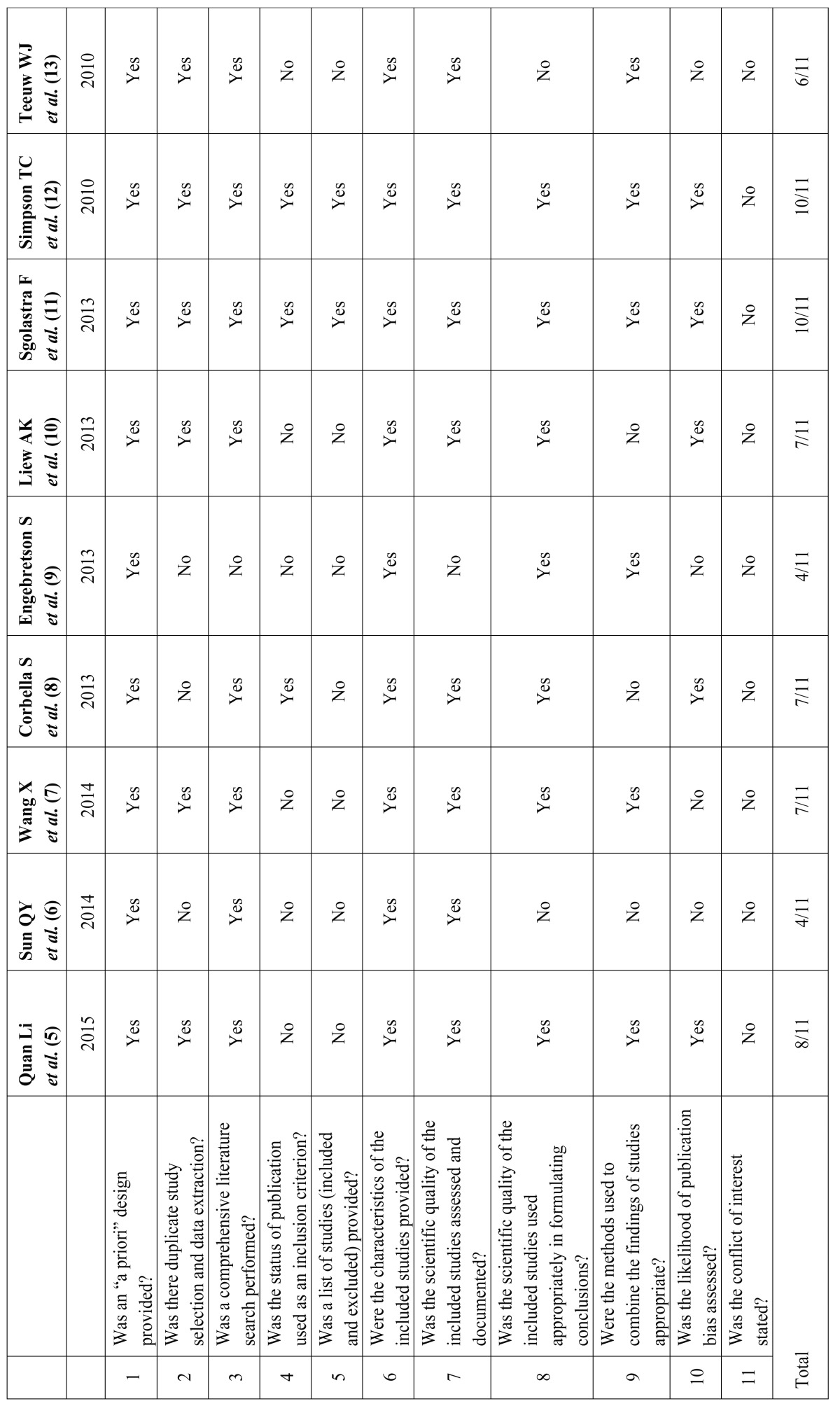
Quality assessment according the AMSTAR items for each SR and total AMSTARs.

## Discussion 

Our SR of SRs reviewed overall evidence supporting the effect of periodontal treatment on diabetes outcomes from the viewpoints of effect size and rigor of the evidence. We can conclude that there is a significant effect of periodontal treatment on improvement of HbA1c in diabetes patients, although the effect size is extremely small. In addition to this small effect size, the supporting evidence cannot be regarded as high quality. These facts regarding periodontitis and diabetes should be well known to healthcare workers and patients.

One of the desirable properties of high-quality systematic review is rigorousness in selecting, synthesizing, and assessing the quality of the evidence. In addition to these characteristics, regular updating is also essential for a good-quality systematic review. The Grades of Recommendation, Assessment, Development, and Evaluation (GRADE) approach is among the most accepted approaches of today for appraising the evidence and generating recommendations in the fields of generating systematic reviews and clinical practice guidelines (26). The GRADE approach has been adopted by countless societies all over the world and has been used in many healthcare fields. In this SR of SRs, GRADE was used only for Simpson’s Cochrane review to appraise the quality of evidence among the nine included SRs (12). This Cochrane review can be judged as a high-quality review based on an AMSTAR assessment.

In this SR of SRs, nine SRs focused on almost the same clinical questions were included. It is remarkable that only one trial was included in the trials lists of all nine SRs (14). One 6-month, single-masked, multicenter, randomized clinical trial (257 participants in each group) (23) was included in two SRs published in 2014 (7) and 2015 (5). The results of this large RCT showed that glycemic control in patients with type 2 diabetes and moderate to advanced chronic periodontitis was not able to be improved by nonsurgical periodontal treatment. Though this result conflict with ones obtained from most of all other trials, statistical weight of this trial tends to be high due to its large sample size. As many authorities in periodontology suggested in the critical article, readers need to take into account that patients’ characteristics in this trial were different from those in other trials (27).

Nine SRs were published within a 5-year period, and three of the nine SRs were published in the same year, 2012. None of the nine SRs has been updated. Whether more SRs focused on same topic are needed is arguable. Updating some specific rigorous and high- quality SRs regularly seems better than performing new SRs conducted using different methodologies and differing in quality.

In this SR of SRs, the qualities of each SR were assessed using the AMSTAR scoring system. AMSTAR scores for each study were not low as a whole, although some SRs only achieved a score of 4. However, it must be recalled that AMSTAR is only a format evaluation system. In the present SR of SRs, obvious transcription errors between indices presented in the meta-analysis and original trials were found in some SRs. Regardless of how high the scores for these SRs become, they cannot be regarded as high-quality SRs. Furthermore, in AMSTAR, there is no item related to updates. It is important to take these things into account when assessing the quality of SRs using AMSTAR.

The spread of clinical practice guidelines and SRs have changed the ways healthcare workers and patients deal with evidence. It is important to continue to discuss how best to generate, access, and assess evidence.

## Conclusions

There is a significant effect of periodontal treatment on improvement of HbA1c in diabetes patients, although the effect size is extremely small. In addition to this small effect size, the supporting evidence cannot be regarded as high quality.
